# To Physicians

**Published:** 1868-09

**Authors:** 


*To Physicians.*—Prof. Horatio R. Storer will deliver his fourth private course of twelve lectures on the Treatment of the Surgical Diseases of Women, during the first fortnight of December, with illustrative operative instruction, at the Franciscan Hospital for Women, under his charge.

Fee $50, and diploma required to be shown. Certificates of attendance upon the previous courses have now been issued to twenty-nine gentlemen in different parts of the country.

Hotel Pelham, Boston, Sept., 1868.

We take pleasure in acknowledging the receipt of valuable medical works, among which is Aitkin's Practice, second American edition, from the fifth London. Philadelphia: Lindsay & Blakiston. We learn from the preface that, in the present edition the editor has carefully revised his contributions, and added much new material. His additions are equal to about three hundred pages of the London edition. They will be chiefly found under the heads of: Lardaceous Degeneration, Vaccination, Measles, Erysipelas, Typhoid, Relapsing, Yellow and Malarial Fevers, Dysentery, Malignant Cholera, Malignant Pustule, Syphilis, Pathology of the Dietic Diseases, Scurvy, Parasitic Diseases, Rheumatism, Gout, Chronic Bright's Disease, Cancer, Tuberculosis, Diseases of the Nervous System, Diseases of the Heart and Lungs, the Sphygmograph, Pyemia, Diseases of the Digestive Organs, Diseases of the Kidneys, and Diseases of the Cutaneous System. They also include twenty-two new articles upon subjects not treated of, or only incidentally mentioned by the author.

The subjects of Locomotor Ataxy, Glosso-Pharyngeal Paralysis, Aphasia, Dilatation of the Bronchia, the Sphygmograph and its tracings in disease, were introduced into this text-book by the editor in the first American edition (1866.) They were first treated of by the author in the fifth English edition (1868,) and his articles on these disorders are chiefly condensed from those of the editor, with the exception of the one on Dilatation of the Bronchia, which Dr. Aitkin has abridged from Dr. T. G. Stewart's excellent article in the *Edinburgh Medical and Surgical Journal*, December, 1867.

We have also to mention in this connection Cazeaux's Midwifery; a new translation from the seventh French edition, by William R. Bullock, M. D., and also Hodge on Diseases of Women. Other valuable works have been received, all of which will be noticed more at length in our next number.

